# Caffeic Acid Phenethyl Ester Inhibits Basal Lipolysis by Activating PPAR-Gamma and Increasing Lipid Droplet-Associated Perilipin in Mature Rat Adipocytes

**DOI:** 10.1155/2022/6007233

**Published:** 2022-08-30

**Authors:** Nguyen Thi Thu Trang, Wan-Chun Chiu, Yu-Ting Feng, Shu-Lin Hsieh, Do Dinh Tung, Jungshan Chang, Tsorng-Harn Fong

**Affiliations:** ^1^International Ph.D. Program for Cell Therapy and Regeneration Medicine, College of Medicine, Taipei Medical University, Taipei 110010, Taiwan; ^2^School of Nutrition and Health Sciences, College of Nutrition, Taipei Medical University, Taipei 110010, Taiwan; ^3^Research Center of Geriatric Nutrition, College of Nutrition, Taipei Medical University, Taipei 110010, Taiwan; ^4^Department of Early Childhood Care, Kun Shan University, Tainan 710303, Taiwan; ^5^Graduate Institute of Medical Sciences, College of Medicine, Taipei Medical University, Taipei 110010, Taiwan; ^6^Department of High Technology, Saintpaul Hospital, Hanoi 100000, Vietnam; ^7^Department of Internal Medicine, Military Medical University, Hanoi 100000, Vietnam; ^8^Department of Anatomy and Cell Biology, School of Medicine, College of Medicine, Taipei Medical University, Taipei 110010, Taiwan

## Abstract

Abnormal lipolysis is correlated with metabolic syndrome. Caffeic acid phenethyl ester (CAPE), a natural product from honeybee hives, has been reported to improve metabolic syndrome. However, the effects of CAPE on lipolysis and perilipin-1 (the major lipid droplet-associated protein) in mature adipocytes were not clarified. In this study, mature adipocytes were isolated from the epididymal fat pads of male rats and incubated with CAPE to estimate lipolysis by measuring glycerol release. It was found that the basal lipolysis was inhibited by CAPE in a dose- and time-dependent manner. The lipid droplet-associated perilipin-1 and phosphorylated peroxisome proliferator-activated receptor (PPAR) gamma levels increased following CAPE treatment by Western blot analysis. Moreover, a specific PPAR-gamma inhibitor (T0070907) could partly reverse the effect of CAPE on basal lipolysis. Furthermore, treatment of adipocytes with dibutyryl-cAMP (db-cAMP) or isoproterenol (ISO) increased lipolysis, but the drug-induced lipolysis was abrogated by combination treatment with CAPE. The lipid droplet-associated perilipin-1 level was also decreased in the drug-induced groups but increased when combined treatment with CAPE. In conclusion, our results revealed that a decrease in basal lipolysis and an increase in lipid droplet-associated perilipin-1 levels induced by CAPE may be involved in the regulation of lipid metabolism through activation of PPAR-gamma in mature adipocytes.

## 1. Introduction

Several studies have reported that visceral fat tissue and abnormal lipid metabolism are highly correlated with cardiovascular diseases, type 2 diabetes mellitus, and metabolic syndrome [1, 2]. Adipocytes store excess free fatty acids (FFAs) as triacylglycerol (TAG) in lipid droplets [3]. FFAs are released in response to lipolysis stimulation for energy production or re-esterification of TAG. Moreover, FFAs are considered not only as one of the energy sources for cells but also as important signaling molecules in regulating cellular behavior and activities [4]. Therefore, excess FFAs released from adipocytes into the bloodstream induce lipotoxicity, inflammation, insulin resistance, and apoptosis [5–7] in multiple cells in organs or tissues. The lipolysis of adipocytes must be properly regulated to prevent lipid metabolism-related disorders.

It has been well reported that lipid droplet-associated proteins are correlated with lipid storage and participate in regulating lipid metabolism [8]. Lipid droplet-associated proteins located on the surface of lipid droplets play important roles in association with other proteins for commenting dynamic protein-protein interactions, leading to trigger activation or inhibition signaling of lipolysis [9, 10]. Perilipin-1 (PLIN) is the most abundant lipid droplet-associated protein and acts as a gatekeeper protein in governing intracellular lipolysis in adipocytes under basal conditions [11, 12]. PLIN engages and forms a protective barrier to prevent lipases from accessing the lipid-stored droplets, leading to increased TAG storage by decreasing lipase-mediated TAG hydrolysis [13]. It has been well described that phosphorylation of perilipin by cAMP-dependent PKA is essential for the mobilization of fats in adipose tissue in response to hormone stimuli, and then phosphorylated perilipin directly interacts with hormone-sensitive lipase (HSL) to initiate and induce lipolysis [14].

Several hormones and drugs such as epinephrine or antidiuretic peptide, dibutyryl-cAMP (db-cAMP), or isoproterenol (ISO), participate in lipid metabolism and induce lipolysis through various lipolytic pathways [15]. The role of cAMP-dependent protein kinase A (PKA) in lipolytic pathways has been highlighted in most studies, wherein catecholamines bind to beta-adrenoreceptors and activate membrane-bound adenylyl cyclases, thereby increasing intracellular cAMP production. Subsequently, increased cAMP level coupled with enhanced PKA activity contribute to the phosphorylation and activation of HSL and PLIN. Activated HSL and PLIN elicit the hydrolysis of TAG and release of FFAs and glycerol [15, 16].

Peroxisome proliferator-activated receptors (PPARs) are ligand-activated transcription factors of the nuclear hormone receptor superfamily including PPAR-alpha, PPAR-gamma, and PPAR-delta, which are expressed in liver, adipose tissue, and skeletal muscle, respectively [17]. Previous studies indicated that PPAR-gamma enriched in adipocytes is the major regulator protein engaging in adipogenesis. During adipocyte differentiation, activated PPAR-gamma is responsible for the induction of several genes involved in adipocyte function [18]. PPAR-gamma regulates both lipid metabolism and inflammation [19]. In addition, a PPAR-responsive element exists within the PLIN gene; thus, PPAR-gamma can regulate PLIN protein expression [20]. The results from gel mobility shift and chromatin immunoprecipitation assays demonstrated that PPAR-gamma could bind to the promoter of the PLIN gene and then subsequently elevate the PLIN level [21]. Two different PPAR-gamma activators (including darglitazone and rosiglitazone) could induce the expression of PLIN in adipocytes [22]. Therefore, it is suggested that PPAR-gamma regulates the expression of lipid droplet-associated PLIN.

Caffeic acid phenethyl ester (CAPE) is an active component of propolis extract; it has a range of biological activities including antimicrobial, antioxidant, anticancer, and anti-inflammatory properties [23, 24]. Several studies have demonstrated that CAPE can regulate lipid metabolism. CAPE significantly inhibits TAG accumulation in cultured 3T3-L1 preadipocytes and then suppresses 3T3-L1 differentiation to adipocytes [25]. In addition, CAPE improves metabolic syndrome and obesity by inducing PPAR-gamma activation and adipose tissue remodeling [26, 27]. According to previous studies, significantly increased basal lipolysis was observed in individuals who suffered from obesity and insulin resistance [28]. However, the roles of CAPE on basal lipolysis remain unclear and the functional correlations between CAPE and lipid droplet-associated proteins in mature adipocytes are required to be explored and clarified.

Defects in lipid metabolism are highly correlated with several diseases. Previous studies suggested that CAPE might prevent obesity and improve metabolic syndrome, so we investigated the effects of CAPE on lipolysis in mature adipocytes under either basal or drug-stimulated conditions. Due to the important roles of PPAR-gamma and its regulated PLIN in fatty acid storage, energy consumption, and obesity, we characterized their roles and molecular correlations among CAPE, PPAR-gamma, and PLIN in lipolysis in mature adipocytes.

## 2. Materials and Methods

### 2.1. Reagents and Chemicals

The CAPE can be enzymatically synthesized by reacting caffeic acid with phenethyl alcohols [29, 30]. In order to improve the purity and stabilize the quality, we purchased synthetic caffeic acid phenethyl ester (CAPE) from Sigma–Aldrich (St. Louis, MO, USA). The final product is an off-white powder. The purity of CAPE is greater than 97% according to the HPLC assay. Because the solubility of DMSO is better than that of ethyl acetate (50 mg/mL), we dissolved the CAPE in DMSO for this study. Moreover, dibutyryl-cAMP (db-cAMP), isoproterenol (ISO), collagenase type II, free glycerol determination kit, dimethyl sulfoxide (DMSO), anti-phospho-PPAR-gamma (pSer112), and anti-PPAR-gamma antibodies were also purchased from Sigma-Aldrich (St. Louis, MO, USA). The PLIN polyclonal antibody was purchased from ThermoFisher Scientific (Waltham, MA, USA). Biotinylated anti-mouse or anti-rabbit IgG antibodies were purchased from VECTOR (Burlingame, CA, USA). Dulbecco's modified Eagle's medium (DMEM), a high glucose medium, was purchased from GIBCO (Grand Island, NY, USA). The materials for sodium dodecyl sulfate-polyacrylamide gel electrophoresis (SDS-PAGE) were purchased from Bio-Rad (Hercules, CA, USA). An ABC kit, which enhances the Western blot signal, was purchased from Vector Laboratory (Burlingame, CA, USA). The XTT Cell Viability Kit for cell viability assay was purchased from Cell Signaling Technology (Danvers, MA, USA).

### 2.2. Animal

We purchased male Sprague–Dawley (SD) rats from BioLASCO Taiwan Co. (Yilan, Taiwan). The adult rats used in the experiments weighed approximately between 500 and 600 g. The rats were housed under a 12 : 12 h light/dark daily cycle at 23°C, and they were fed chow and water ad libitum under standard laboratory conditions. All animal care procedures were approved by the Taipei Medical University Ethical Committee for Laboratory Animals and performed in accordance with the guidelines of the committee. All protocols were approved by the Institutional Animal Care Committee, Taipei Medical University (No: LAC-2019-0080).

### 2.3. Preparation and Incubation of Isolated Mature Adipocytes

Rats (*n* = 20) were injected intraperitoneally with sodium pentobarbital at a dose equivalent to 40 mg per kg of body weight for anesthesia before euthanasia. The procedure described by Rodbell with some modifications to isolate adipocytes was performed [31]. First, the epididymal fat pads were removed immediately. Next, the fat pads were incubated at 37°C and washed three times in a DMEM medium. The fat pads were minced with scissors and mixed with a medium that contained Type-II collagenase (3.3 mg/mL) to digest the collagen in the fat pads through constant mild shaking for 60 min at 37°C. Subsequently, the adipocytes were isolated by a nylon mesh and centrifuged for 5 min at 1000 rpm. The supernatant layer containing fat cells was collected and washed three times with DMEM medium to eliminate collagenase and then resuspended in DMEM containing 2.5% bovine serum albumin (BSA) at a density of 1 × 10^5^ cells/mL. Next, this cell mixture was incubated with CAPE db-cAMP or ISO at 37°C for 30, 60, 90, and 120 minutes.

### 2.4. Measurement of Cell Viability

The XTT Cell Viability Kit was used to determine the cytotoxicity of CAPE according to the manufacturer's protocol. Adipocytes were treated with 100 *μ*M CAPE at multiple time points before an XTT assay was performed. After CAPE treatment, the cell mixture was washed with DMEM and centrifuged at 200 g for 3 min. The floating adipocytes were collected and diluted with DMEM. The optimized number of cells (about 5 × 10^3^ cells) for the assay was placed into the wells of a flat-bottom 96-well plate in triplicate and added 50 *μ*L of the activated-XTT solution to each well. The plate was returned to a CO_2_ incubator at 37°C for 2 h. The absorbance at 450 nm in each well was measured, recorded, and analyzed.

### 2.5. Measurement of Glycerol Release

After drug treatment, the tubes containing adipocytes and glycerol were shaken and centrifuged at 200*g* for 3 min. The fat cells floating on top were collected for SDS-PAGE or isolation of intracellular lipid droplets, and the lower medium was collected and measured for its glycerol content with a Free Glycerol Determination Kit which contains glycerol kinase and glycerol phosphate oxidase. 400 *μ*L of glycerol kit was mixed with 25 *μ*L of medium in each tube. The absorbance at 540 nm indicated the occurrence of glycerol release in accordance with the manufacturer's instructions.

### 2.6. Isolation of Intracellular Lipid Droplets and SDS-PAGE

Intracellular lipid droplets were isolated from fat cells in accordance with the method used by Okuda et al. (with minor modifications) [32]. Specifically, after the completion of drug incubation, the fat cells were washed twice with normal saline to remove BSA from the reaction medium and then incubated in lysis buffer (5 mM Tris buffer, 7.4 pH, 0.025% Triton X-100, 1 mM EDTA, 50 mM NaF, 10 *μ*g/mL leupeptin, and 1 mM benzamidine) on ice for 15 min. The samples were vortexed and then centrifuged at 13,000*g* for 15 min at 4°C. The floating fat cake fractions were collected and mixed with equal volumes of 2 × sample buffer (62.5 mM Tris, 6.8 pH, 5% beta-mercaptoethanol, 2% sodium dodecyl sulfate, and 10% glycerol). The cell lysate samples were directly mixed with equal volumes of 2 × sample buffer after drug treatment. The samples were heated to 95°C for 5 min and clarified by centrifugation at 10,000 rpm for 5 min before they were used for SDS-PAGE. Equal amounts of the samples were loaded and resolved through SDS-PAGE on 10% polyacrylamide slab gels.

### 2.7. Western Blot Analysis

Intracellular lipid droplets were used to detect PLIN, and cell lysates were used to detect PPAR-gamma. We used 10% SDS polyacrylamide gel to run a Western blot that corresponds to the molecular weight of the target proteins. Subsequently, the proteins were transferred onto polyvinylidene difluoride (PVDF) membranes by operating a semidry transfer cell machine at 20 V for 40 min. Then 5% nonfat milk in Tris-buffered saline (TBS; 50 mM Tris and 200 mM NaCl, pH 7.5) containing 0.1% Tween-20 was used as a blocking solution at room temperature for 1 h on the shaker. Primary antibodies were diluted with TBS according to the manufacturer's instructions. The PVDF membranes with the primary antibodies were incubated overnight at 4°C after washing with TBS three times for 10 min per wash. The PVDF membranes were incubated with a secondary antibody (biotinylated anti-mouse or anti-rabbit IgG) at room temperature for 2 h without shaking. After three 10-min washes with TBS were performed, the reactive bands were enhanced through incubation with the ABC kit, which enhanced the signal at room temperature for 1 h. After a 10-min wash with TBS, the reactive bands were visualized using an enhanced chemiluminescence method.

### 2.8. Statistical Analysis

The means and standard errors of the mean for three or more experiments were obtained. The Student's *t*-test or analysis of variance (ANOVA) was used to compare the experiment and control groups. A *P* value of < 0.05 indicated a significant difference.

## 3. Results

### 3.1. CAPE Inhibited Glycerol Release in a Dose- and Time-Dependent Manner

**Figure 1:**
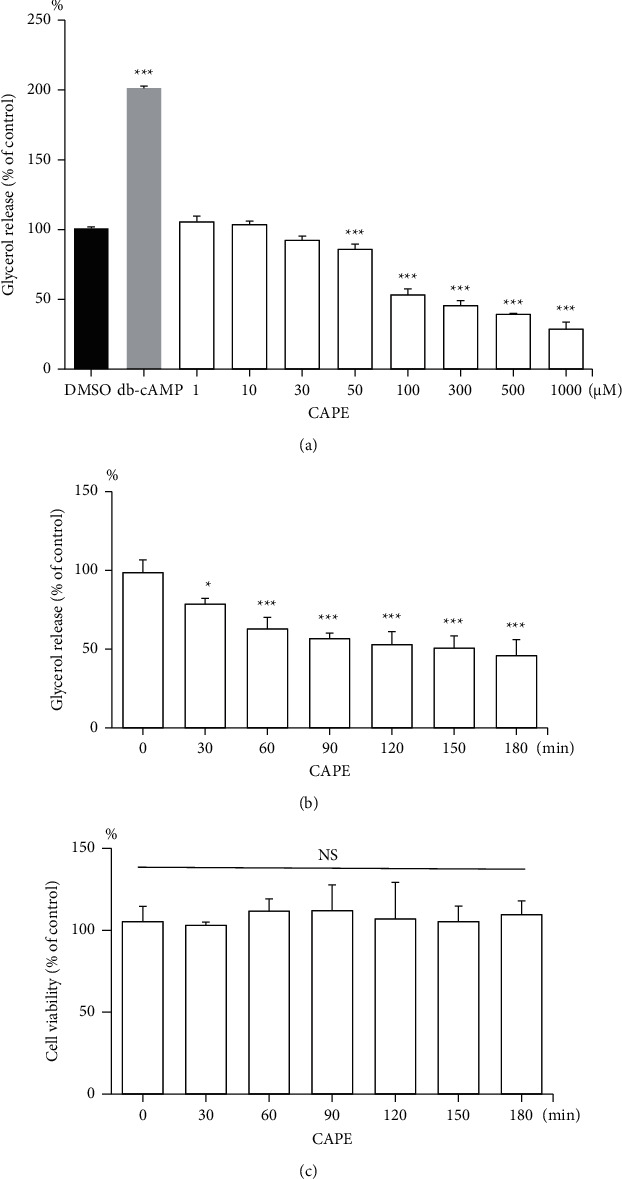
CAPE treatment inhibits glycerol release in a dose- and time-dependent manner in mature adipocytes. (a) Adipocytes were treated with DMSO (0.1%), db-cAMP (1 mM), or CAPE (1, 10, 30, 50, 100, 300, 500, and 1000 *μ*M) for 2 h. The db-cAMP-treated group served as the positive control for glycerol release. (b) Adipocytes were treated with CAPE (100 *μ*M) for 0, 30, 60, 90, 120, 150, and 180 min. (c) Cell viability was determined through an XTT assay. Adipocytes were incubated with 100 *μ*M CAPE multiple times compared with the control (0-min) group and exhibited no cytotoxicity. Data are expressed as percentages of the control DMSO group or 0-min group and are presented as means ± standard errors (*n* = 3); ^*∗*^*P* < 0.05 and ^*∗∗∗*^*P* < 0.001 compared with the control DMSO group or 0-min group. NS: no statistical difference.

To investigate the effects of CAPE on basal lipolysis of mature rat adipocytes, glycerol releases following various doses (from 1 *μ*M to 1000 *μ*M) of CAPE treatments for 2 h were examined. As shown in Figure 1(a), CAPE inhibited glycerol release in a dose-dependent manner. Low-dose CAPE (1 *μ*M, 10 *μ*M, or 30 *μ*M) had no significant effect on glycerol release compared with the control (0.1% DMSO) group. Compared with the control group, the glycerol release in the experimental group significantly decreased to 85.9 ± 3.6%, 53.3 ± 4.2%, 45.5 ± 3.6%, 39.3 ± 0.6%, and 28.7 ± 5.0% following treatment with 50, 100, 300, 500, and 1000 *μ*M CAPE, respectively (*P* < 0.001). Db-cAMP (1 mM), a lipolytic agent, was used as the positive control to increase glycerol release by 201.3 ± 1.5% relative to the control group (*P* < 0.001) (Figure 1(a)). This result revealed that CAPE dose-dependently inhibited basal lipolysis in mature adipocytes. After treatment with 100 *μ*M CAPE, the measured glycerol release of the experimental group was approximately half that of the control group. Therefore, 100 *μ*M CAPE in the subsequent experiments was used.

Furthermore, to study the effects of CAPE on lipolysis at various time points, the glycerol release of adipocytes treated with 100 *μ*M CAPE at 0, 30, 60, 90, 120, 150, and 180 min was measured.  Figure 1(b) shows that CAPE (100 *μ*M) reduced glycerol release in a time-dependent manner. Relative to the 0-min group, glycerol decreased to 78.6 ± 3.6% (*P*=0.045), 62.9 ± 7.2%, 56.6 ± 3.6%, 52.9 ± 8.2%, 50.6 ± 7.8%, and 45.8 ± 10.1% (*P* < 0.001) following treatment with CAPE (100 *μ*M) for 30, 60, 90, 120, 150, and 180 min, respectively (Figure 1(b)). The results indicated that CAPE (100 *μ*M) time-dependently reduced the glycerol release of mature adipocytes within 3 h.

Furthermore, the cell viability results from the XTT assay indicated that the CAPE did not exert cytotoxic effects. Cell viability of adipocytes post-treated with 100 *μ*M CAPE was monitored and compared every 30 minutes for 180 minutes. There are no significant changes in cell viability (Figure 1(c)). This result demonstrated the reduction in glycerol release was not associated with cytotoxicity of adipocytes.

### 3.2. CAPE Increased Lipid Droplet-Associated PLIN and Phosphorylation of PPAR-Gamma Levels

**Figure 2:**
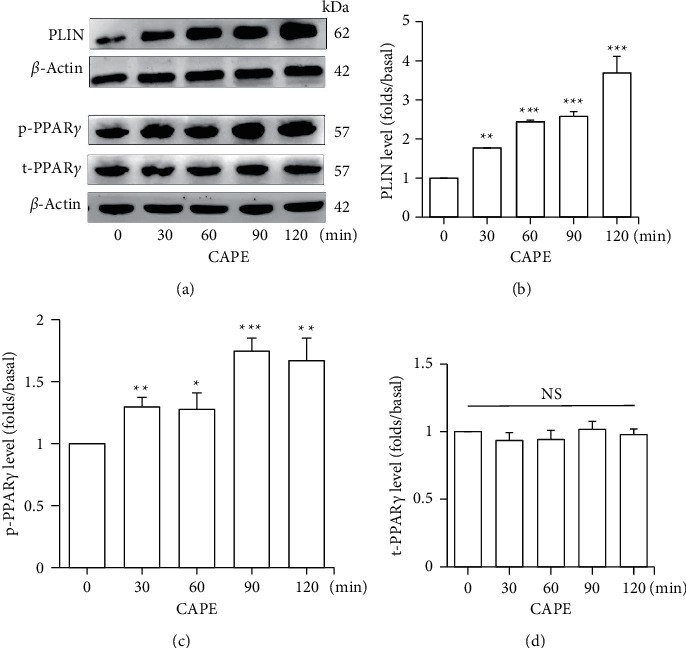
Effects of CAPE on PLIN, phospho-PPAR*γ*, and total-PPAR*γ* in mature adipocytes. Adipocytes were incubated with CAPE (100 *μ*M) for the indicated time period. (a) PLIN (62 kDa), phospho-PPAR*γ* (57 kDa), total-PPAR*γ* (57 kDa), and *β*-actin (42 kDa) were detected using a Western blot, (b) lipid droplet-associated PLIN, (c) phospho-PPAR*γ*, and (d) total-PPAR*γ* were quantified as ratios to *β*-actin in each group. Data are expressed as means ± standard errors of three separate experiments (*n* = 3); ^*∗*^*P* < 0.05, ^*∗∗*^*P* < 0.01 and ^*∗∗∗*^*P* < 0.001 compared with the 0-min group. NS: no statistical difference.

PLIN is an essential regulator of lipid droplet stability and is involved in lipolysis. As shown in Figure 2(a), the level of lipid droplet-associated PLIN (62 kD) increased gradually following CAPE treatment for 120 minutes, whereas the level of beta-actin (42 kD) did not change. The quantitative data indicated that the lipid droplet-associated PLIN level increased 1.8-, 2.4-, 2.6-, and 3.7-fold relative to the basal (0-min) group after incubation with CAPE for 30, 60, 90, and 120 min, respectively (Figure 2(b)). CAPE treatment time-dependently increased the lipid droplet-associated PLIN level of mature adipocytes.

In addition, PPAR-gamma plays a key role in regulating lipid metabolism in adipose tissue. As shown in Figures 2(a) and 2(c), the phosphorylation form of PPAR-gamma increased 1.3-, 1.3-, 1.7-, and 1.7-fold relative to the basal (0-min) group after CAPE treatment for 30, 60, 90, and 120 minutes, respectively. The change in the total level of PPAR-gamma during CAPE treatment was not significant (Figures 2(a) and 2(d)). The data suggested that phosphorylation of PPAR-gamma is involved in the antilipolytic pathway of CAPE in mature adipocytes.

### 3.3. PPAR-Gamma Inhibitor (T0070907) Attenuated the Effects of CAPE on Basal Lipolysis and Lipid Droplet-Associated PLIN Level

**Figure 3:**
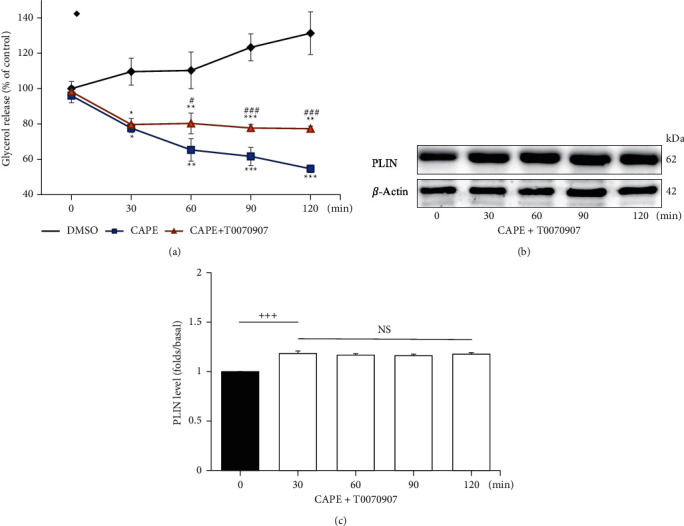
Effects of PPAR-gamma inhibitor (T0070907) on glycerol release and PLIN level in CAPE-treated adipocytes. Adipocytes were incubated with 0.1% DMSO, 100 *μ*M CAPE, or a combination of CAPE with a 10 nM PPAR-gamma inhibitor (T0070907) for the indicated periods. (a) Glycerol release was measured in each group. (b) Lipid droplet-associated PLIN (62 kDa) and *β*-actin (42 kDa) were detected using a Western blot. (c) Lipid droplet-associated PLIN levels were quantified as ratios to *β*-actin in each group. Each point represents the mean ± standard error of three separate experiments (*n* = 3). ^*∗*^*P* < 0.05, ^*∗∗*^*P* < 0.01, and ^*∗∗∗*^*P* < 0.001 compared with the DMSO-treated group. ^#^*P* < 0.05, ^##^*P* < 0.01, and ^###^*P* < 0.001 compared with the CAPE-treated group. ^+++^*P* < 0.001 compared with the 0-min group. NS: no statistical difference.

To clarify the contribution of PPAR-gamma in the antilipolytic pathway of CAPE, we used the drug T0070907 (a potent and selective PPAR-gamma inhibitor) in the subsequent experiment.  Figure 3(a) shows that the glycerol release slowly accumulated and increased in the DMSO (0.1%) group for 120 min. CAPE (100 *μ*M) alone significantly inhibited glycerol release in a time-dependent manner. However, compared with the control (0-min) group, the CAPE–T0070907 (10 nM) treatment reduced glycerol release to 79.7 ± 3.5%, 80.3 ± 5.9%, 77.7 ± 2.1%, and 77.3 ± 1.5% at 30, 60, 90, and 120 min, respectively. A significant decrease in glycerol release was observed at 30 min compared with the control (0-min) group (*P*=0.035), but statistical differences were not evident between 30, 60, 90, and 120 min.

By contrast, the lipid droplet-associated PLIN level also increased following CAPE–T0070907 treatment for 30 min but remained stable thereafter (Figures 3(b) and 3(c)). The data suggested that coincubation with a PPAR-gamma inhibitor attenuates the inhibitory effect of CAPE on glycerol release and lipid droplet-associated PLIN levels.

### 3.4. CAPE Inhibited Db-cAMP-Induced and ISO-Induced Lipolysis by Increasing Lipid Droplet-Associated PLIN Level

**Figure 4:**
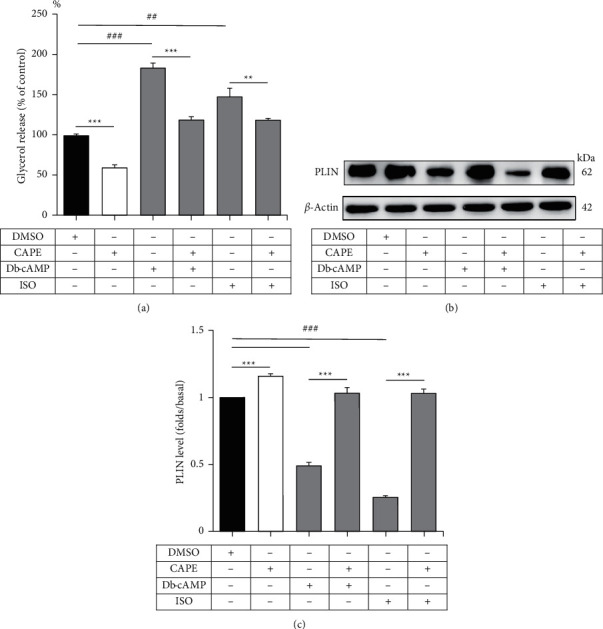
CAPE inhibits db-cAMP-induced or ISO-induced glycerol release by increasing lipid droplet-associated PLIN levels in mature adipocytes. Adipocytes were incubated with db-cAMP, ISO, or a combination of CAPE with db-cAMP or ISO. (a) Glycerol release was measured in each group. (b) Lipid droplet-associated PLIN (62 kDa) and *β*-actin (42 kDa) were detected using a Western blot. (c) Lipid droplet-associated PLIN levels were quantified as ratios to *β*-actin in each group. Data are expressed as means ± standard errors of three separate experiments (*n* = 3). ^*∗∗*^*P* < 0.01 and ^*∗∗∗*^*P* < 0.001 compared with the non-CAPE-treated group. ^##^*P* < 0.01 and ^###^*P* < 0.001 compared with the DMSO group.

Db-cAMP (1 mM) and ISO (1 *μ*M) can induce glycerol release in adipocytes. To investigate the effects of CAPE on db-cAMP-induced or ISO-induced lipolysis, we combined CAPE with db-cAMP or ISO to treat adipocytes and measured the resulting glycerol release. After 2 h of incubation, glycerol release decreased to 58.9 ± 3.7% in the CAPE-treated group compared with the DMSO group (*P* < 0.001). Compared with the DMSO group, glycerol release increased to 183.0 ± 6.1% (*P* < 0.001) and 147.2 ± 10.6% (*P* < 0.01) in the db-cAMP-treated and ISO-treated groups, respectively. However, compared with the DMSO group, glycerol release decreased to 118.3 ± 4.1% and 118.2 ± 2.1% in the CAPE with db-cAMP and CAPE-with-ISO groups, respectively (Figure 4(a)). These findings suggested that CAPE suppresses db-cAMP-induced and ISO-induced glycerol release.

In addition, the lipid droplet-associated PLIN level in db-cAMP and ISO in the absence or presence of CAPE was examined through Western blot analysis. As shown in Figures 4(b) and 4(c), lipid droplet-associated PLIN levels increased 1.2-fold in the CAPE-only group compared with the DMSO group (*P* < 0.001), but they decreased 0.5-fold and 0.3-fold in the db-cAMP and ISO groups, respectively, compared with the DMSO group (*P* < 0.001). The lipid droplet-associated PLIN levels in the CAPE with db-cAMP and CAPE-with-ISO groups were not significantly different from those in the DMSO group. The results suggest that CAPE increases lipid droplet-associated PLIN levels and reduces glycerol release when combined with db-cAMP and ISO.

## 4. Discussion

The results of this study suggest that the incubation of mature adipocytes with CAPE inhibits basal lipolysis in a dose- and time-dependent manner. No cytotoxicity of CAPE on adipocytes was found by using the XTT assay, suggesting that the reduction in glycerol release was not associated with cytotoxicity of adipocytes. To dissect the molecular mechanism, the roles of PPAR-gamma and PLIN in CAPE-mediated lipolysis inhibition were assessed. A significantly increased amount of the phosphorylated form of PPAR-gamma coupled with PLIN expression was detected in rat mature adipocytes incubated with CAPE for various times. To confirm the role of PPAR-gamma on basal lipolysis of mature adipocytes, a specific PPAR-gamma inhibitor (T0070907) was applied. The results indicated the coincubation of CAPE with the PPAR-gamma inhibitor (T0070907) reversed the antilipolytic effect of CAPE on glycerol release and lipid droplet-associated PLIN levels. In addition, the incubation of mature adipocytes with db-cAMP or ISO reduced PLIN levels on the lipid droplets and then enhanced lipolysis. However, the administration of CAPE inhibited either db-cAMP- or ISO-induced glycerol release by increasing lipid droplet-associated PLIN production. Taken together, these results suggested that CAPE-induced phosphorylation of PPAR-gamma increased lipid droplet-associated PLIN production resulting in the suppression of lipolysis of rat mature adipocytes.

The role of CAPE on adipogenesis of preadipocytes has been previously described. It has been reported that TAG accumulation in 3T3-L1 murine preadipocytes treated with CAPE at concentrations of 25–50 *μ*M for 2 days was significantly inhibited and 3T3-L1 differentiation was also suppressed to form mature adipocytes [25]. Another study indicated CAPE at 40 *μ*M almost blocked lipid accumulation during the early stage of adipogenesis (days 0−4) [27]. These studies suggested CAPE at low concentrations (20–50 *μ*M) for a long time (2–4 days) could inhibit adipogenesis by reducing the accumulation of TAG, leading to the inhibition of the differentiation of 3T3-L1 preadipocytes into mature adipocytes. However, the biological role of CAPE was evaluated on mature adipocytes from rat epididymal fat pads instead of preadipocytes in our current study, and the results revealed that rat mature adipocytes treated with CAPE at a higher concentration (100 *µ*M) for a shorter duration (3 hours) displayed a significant inhibitory effect on glycerol release. Besides, neither adipogenesis nor cytotoxicity was observed in CAPE-treated mature adipocytes.

PPARs, a set of fatty acid-regulated transcription factors, are involved in regulating lipid metabolism and inflammation [19]. The role of PPAR-gamma in CAPE treatment remains controversial. It has been demonstrated that CAPE improves insulin sensitivity and reduces lipopolysaccharide-induced inflammatory mediators by increasing PPAR-gamma levels [33]. A study reported that the activation of PPAR-gamma induced by CAPE could improve metabolic syndrome and remodel adipose tissue in obese mice induced by a high-fat diet [26]. However, another study demonstrated that CAPE could suppress PPAR-gamma, C/EBP-alpha, Fas, and aP2 during the 3T3-L1 differentiation process [25]. Our study discovered that CAPE treatment induced phosphorylation of PPAR-gamma in mature adipocytes. In addition, the antilipolytic effect of CAPE was reversed by a specific PPAR-gamma inhibitor (T0070907) in mature adipocytes. These results indicated that CAPE activated PPAR-gamma via phosphorylation, leading to an increase in lipid droplet-associated PLIN protein level coupled with a reduction in basal glycerol release.

Furthermore, PLIN has been suggested as one of the PPAR-gamma downstream targeting gene products in adipocytes. PLIN expression is highly correlated with PPAR-gamma activation in adipocytes [22]. PPAR-gamma is involved in the transcriptional regulation of the perilipin genes through the PPAR-responsive element within the perilipin gene [20]. Treatment of 3T3-L1 adipocytes with agonists of PPAR-gamma significantly enhanced perilipin gene expression in fully differentiated adipocytes [21]. The activation of PPAR-gamma enhanced mitochondrial biogenesis, upregulated the expression of PLIN, and induced multilocularization in mouse mature adipocytes [34]. Our data also indicated that CAPE treatment increased PPAR-gamma phosphorylation and enhanced PLIN levels in rat mature adipocytes.

PLIN, which is localized on the surface of lipid droplets, participates in the stabilization of intracellular lipid droplets and regulation of lipolysis [35]. PLIN expression is essential in the hormonal regulation of lipolysis in adipose tissue [12]. Significantly lower PLIN levels were detected in the abdominal subcutaneous adipose tissue of individuals suffering from severe obesity compared to normal ones with physical fitness [36]. Reduced PLIN expression was observed in women with obesity compared with women without obesity [37]. Moreover, PLIN knockdown (PLIN ^–/–^) mice are much leaner and have smaller white adipocytes than wild-type mice. Isolated adipocytes from PLIN ^–/–^ mice exhibit elevated basal lipolysis and reduced lipid storage because of the loss of the protective function of PLIN [38]. TAG hydrolysis is accelerated, leading to an approximate 70% reduction in adipose tissue mass in PLIN null mice [39, 40]. These studies support the concept that PLIN promotes TAG storage under basal conditions at least by reducing the access of cytosolic lipases to TAG stored in lipid droplets. In our study, we revealed that CAPE treatment could increase lipid droplet-associated PLIN levels and decrease basal lipolysis or drug-stimulated lipolysis in mature adipocytes. Our data could support that increasing PLIN could better protect fat droplets and reduce basal lipolysis.

It has been reported that higher basal lipolysis was observed in obese people compared to people in trimness shape in the previous studies [28, 36]. In addition, studies with antidiabetic drugs such as troglitazone and metformin have indicated that the inhibition of basal lipolysis is a common mechanism for improving insulin sensitivity in adipocytes [41, 42]. Lipolysis of adipose tissue is dysregulated in obesity, insulin resistance, and cancer cachexia [43]. Thus, the partial inhibition of basal lipolysis may be a potential therapeutic strategy for preventing metabolic disorders.

**Figure 5:**
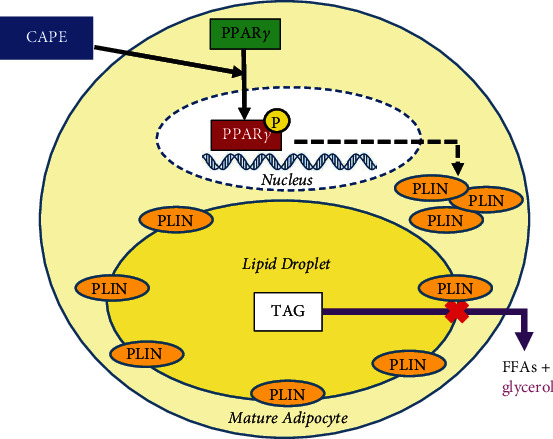
Schematic model of CAPE on lipid mobilization. CAPE inhibited lipolysis (glycerol release) through phosphorylation of PPAR-gamma and increased PLIN production in mature adipocytes.

In conclusion, the study evidenced that CAPE, a natural product of propolis from honeybee hives, suppressed basal lipolysis by increasing PPAR-gamma phosphorylation and the lipid droplet-associated PLIN levels in rat mature adipocytes (as illustrated in Figure 5). This result may contribute to the verification of the involvement of CAPE in basal lipolysis and the development of improved obesity and metabolic syndrome therapy in the future.

## Data Availability

The data used to support the findings of this study are available from the corresponding author upon request.
